# Morbid Anatomy

**Published:** 1836-10

**Authors:** 


### PART THIRD.

### Selections from Foreign Journals.

### MORBID ANATOMY.

### *Case of Aneurism of the Thoracic Duct.* By Dr. Albers, of Bonn.

The patient, a man of fifty-one, died of abscess of the liver. On examining the body after death, Dr. Albers found, in the region of the solar plexus, amongst several hard cartilaginous tumours, an elastic soft one, with a half-transparent tegument. It was knotty, and about the shape of a fig. At first he took it for an hydatid; but it was soon discovered that it was bound by membranous bands in several places, and that a canal led into it, both above and below. It contained a quantity of fluid lymph, in which flaky matter was suspended. The internal surface of the tumour was smooth and uniform. A sound could be passed up the canal, both superiorly and inferiorly. In the latter direction its course was followed, and it soon became evident that the tumour was an aneurism of the thoracic duct. Its parietes were thicker and firmer than those where the duct had not lost its normal caliber.

Dr. Albers has only seen one similar case, viz. a dilatation of the cysterna chyli, found at the examination of a dropsical patient.

It is singular that the thoracic duct is not oftener affected by the diseases of the surrounding organs. How often it is compressed in the scrofulous and consumptive, by enlarged glands, tumours, &c.! But these compressions are not attended by corresponding dilatations, as is the case with arteries and veins. Dr. A. has seen a case in which the thoracic duct, in the middle of its course, had been reduced by pressure to such a small caliber, that it would not admit even a bristle. In another case the canal was altogether obliterated; but in neither was the caliber of the canal below the narrowed part altered by the compression. (See also Rokitanski, *Austrian Annals*, vol. xvii. p. 441.) The cause of this absence of dilatation in cases of compression lies, doubtless, in the nature of the fluid, in the weakness of its current, and also in the fact that there must be more branches of the thoracic duct anastomosing with the venous system than is generally supposed. A proof of this is, that in children in whom the caliber of the duct has been materially narrowed, no emaciation has followed in consequence. Wutzer has discovered a branch of the thoracic duct leading into the vena azygos. The most frequent cases of dilatation of lymphatic vessels are those in which they contain tuberculous, scrofulous, and cancerous matters. Numerous descriptions of such cases are found in the works of Cruveilhier and Carswell.

*Hannoversche Annalen*, B. ii. H. 1. 1836.

### *On the Anatomical Condition of Diseased Bone.* By Professor Gerdy, Surgeon of the Hospital St. Louis.

In a previous essay, the author published his views on the normal structure of bone; without a correct acquaintance with which, he considers that there can be no accurate knowledge of their morbid conditions. A note appended to the present essay contains the following outline of his opinions on this subject.

Bones consist of—1. The *compact tissue;* composed of very fine and compressed bony tubes, lying parallel to the axes of the long bones, and sometimes in the flat bones converging towards the original centres of ossification. The furrows which are seen on the surface of long bones and of some of the flat bones, penetrate frequently into the little canals of the compact tissue, and even into the diploë. 2. The *canaliculated tissue* is that which is inappropriately termed spongy tissue. It is an assemblage of little longitudinal canals, perforated by apertures in their parietes, so that they communicate with one another. 3. The *interrupted canaliculated tissue.* Here the canals are so interrupted by laminæ of bone, that the appearance would justify the terms areolar or cellular. This tissue occupies the epiphyses of long bones, short bones, and the interval of the laminæ of flat bones. 4. The *reticular tissue* is a network of solid bony fibres, occupying the medullary cavity of the long bones, and of such of the short bones as possess these cavities. The vessels consist of very delicate plexuses, with extremely thin parietes, spreading through all the little canals, and anastomosing through the apertures which have been mentioned, and in the midst of the medulla which envelopes them. The medulla is a cellulo-adipose tissue, of the same nature in the medullary and in the canaliculated cavities: it is simply less in quantity in the very fine vascular canals than in the larger ones, and than in the medullary cavities in particular.

After this brief sketch of the structure of bone, the author proceeds to state that osteitis is of much more frequent occurrence than is generally believed; that all the injuries and most of the diseases of bones are attended with inflammation; and that the existence of such inflammation is demonstrable from the permanence of its effects. This inflammation is rarely simple, the periosteum or cellular tissue connecting the bones with their cartilages being generally implicated in the same diseased action. Osteitis may exist in all the bony tissues; and, whichever may be primarily affected, the inflammation rapidly spreads to the others. Inflamed bones are swollen; sometimes, with loss of weight, at others with an increase of it. They are never found softer than in the healthy state; and the author believes that their fragility has given rise to the idea that they become softened by inflammation. The changes in their structure, from inflammation, are of three kinds.

1. *The rarefying osteitis* in which the bones become lighter and more fragile. This consists in a dilatation of the furrows and of the little canals, and of their vascular apertures. When a bone is universally inflamed, its chief nourishing canal (supposing it to possess one,) acquires double its normal size, and sometimes this canal is extended as an isolated tube in the interior of the bone, as if it were secreted by the artery and vein which it encloses. All the tissues of the bone become dilated. When, by maceration, the soft parts are separated, the osseous portion which remains is as hard as in healthy bone. The distended canals in the interior of the bone are filled with a red medulla, the vessels of which appear to be augmented both in number and size.

2. *The condensing osteitis.* In this form of inflammation, all the tissues become thickened and more dense; but it is occasionally combined with the previous condition; the bone being in some parts more dense, in others more dilated, than in the healthy state. When cut, the compact tissue appears like a piece of stone which has been sawed; a greater degree of condensation is noticed in the other tissues, and sometimes the medullary canal of the long bones is obliterated by a deposition of stony hardness.

3. *The ulcerating osteitis,* or caries, is accompanied by more or less suppuration. This inflammation is superficial, and presents a rough, worm-eaten surface, or deep sinuous and rugged ulceration. Hitherto the nature of these conditions has not been recognized. The roughness and worm-eaten structure are dependent on a morbid increase of size of the openings in the vascular furrows, and on the formation of irregular apertures by absorption of the bony tissue. Beneath the surface of caries exists an inflammatory condition of the bone, with all the anatomical characters previously described. Splinters of bone, of various form, are also separated by inflammation; and the changes produced by this process are evident in the exfoliations. These splinters have the rarefied structure, quite distinct from that which is seen in the compact tissue of necrosed bone; the processes of caries and necrosis being essentially different. The portions of bone separated by caries are not, as has been lately asserted, destitute of gelatine; but preserve the same form when their calcareous constituents have been dissolved by muriatic acid.

Periosteal inflammation frequently attends on osteitis; the periosteum becoming thickened, losing its adherence to the bone, softened or indurated, and injected by vessels, both more numerous and larger, than in the healthy state. Bony concretions are formed on the surface of the bone itself. These are of various forms and sizes, and are secreted by the inflamed membrane. When osteitis is complicated with inflammation of the medullary tissue, (and by this term is meant the whole adipose tissue of bones,) the medulla is either partially or entirely red and inflamed; the colour varying in depth: it may be either softened or hardened, and in some cases has an ecchymosed appearance, or contains small purulent depositions. When the bone is inflamed at its articular portions, and is accompanied with diarthrodial inflammation, the cellular tissue uniting the cartilages to the bone, and which is invisible in the normal state, becomes considerably thickened; the cartilages become absorbed, partially or entirely, and sometimes perforated, so as to allow the thickened cellular tissue connecting them with the bone to project through these apertures. When the cartilage is entirely removed, the articular extremity of the bone appears to be covered by a thick, velvety, tomentose membrane, of a reddish-brown or grey colour.

In osteitis with hyperostosis, (commonly termed exostosis,) there is partial or general increase of the size and thickness of the bone; and its structure may either be more compact or more dilated than in the normal state; and some portions of it may be the seat of the ulcerative inflammation. Some of the compact hyperostoses may, however, arise independently of inflammation, and depend simply on considerable increase of nutrition. Within the dilated structure of partial exostoses, there occasionally form large cavities, occupied by accidental morbid products, such as encephaloid or colloid matter, which have a great tendency to degenerate into cancer,—that is to say, to become inflamed, soften, ulcerate, suppurate, and to assume all the symptoms characterizing cancer. Various other morbid tissues are formed in these partial exostoses. Their first effect is to produce absorption of the bony matter, and this may go on until the periosteum alone limits their extent. This membrane, being irritated, secretes more bony matter around the morbid formation. From these changes arise the cellular exostoses and osteo-sarcoma. The author concludes his article by noticing the frequency of osteitis in all wounds of bones, fractures, dislocations, which have remained unreduced; diseases of joints, and in ulcerations which extend almost to the surface of bones, and which leave traces in their substance which are never removed,

*Archives Génerales de Medicine.* Tome x. *Février,* 1836.

---

